# Characteristics of urologists participating in Medicare Advantage networks and implications for prostate cancer care

**DOI:** 10.1093/haschl/qxag172

**Published:** 2026-07-04

**Authors:** Avinash Maganty, Eran Politzer, Asa Hartman, Brent K Hollenbeck, Bruce Landon

**Affiliations:** Department of Urology, Massachusetts General Hospital, Boston, MA 02114, United States; Department of Surgery, Harvard Medical School, Boston, MA 02115, United States; Department of Health Care Policy, Harvard Medical School, Boston, MA 02115, United States; Federmann School of Public Policy and Governance, The Hebrew University of Jerusalem, Jerusalem 91905, Israel; Federmann School of Public Policy and Governance, The Hebrew University of Jerusalem, Jerusalem 91905, Israel; Department of Urology, University of Michigan, Ann Arbor, MI 48109, United States; Department of Health Care Policy, Harvard Medical School, Boston, MA 02115, United States; Division of General Medicine, Beth Israel Deaconess Medical Center, Boston, MA 02115, United States

**Keywords:** Medicare Advantage, urology, prostate cancer, quality

## Abstract

**Background:**

Enrollment in Medicare Advantage (MA) has expanded rapidly and MA plans may rely on selective provider networks to manage utilization. While selective contracting has been described for primary care physicians, less is known about how MA networks are constructed for surgical specialists such as urologists, who manage common and costly conditions including prostate cancer.

**Methods:**

We conducted a national observational study using a 20% sample of Medicare fee-for-service claims (2016 to 2021) linked to 2021 MA network data. We identified 9124 urologists practicing in hospital referral regions (HRRs) with at least 1 MA network. Urologists were classified as in-network or out-of-network within each network HRR and further categorized by network breadth, defined as narrow (<30% of local urologists) or broad. Outcomes were weighted by network enrollment and included urologist characteristics, prostate cancer–related and benign procedure volume, and 2 claims-based quality measures: use of magnetic resonance imaging (MRI) prior to prostate biopsy and consultation with radiation oncology after new prostate cancer diagnosis.

**Results:**

Among 9124 urologists, 98% participated in at least 1 MA network. Across 7516 network–HRR combinations, networks included a mean (SD) of 42 (47) urologists, with inclusion ranging from 9% to 100% of local urologists. Compared with the average urologist excluded from a network in the HRR, the average in-network urologist had a higher annual patient volume (107 vs 95; *P* < .01), performed more prostate biopsies (3.18 vs 2.89 per year; *P* < .01), and performed more prostatectomies (0.48 vs 0.35 per year; *P* < .01), without differences in quality measures. The average urologist in narrow networks had a lower overall procedure volume but were more likely to perform prostatectomy among newly diagnosed patients (21% vs 14%; *P* < .01). Quality patterns were mixed, with higher MRI use prior to biopsy (15% vs 12%; *P* < .01) but lower radiation oncology consultation (50% vs 54%; *P* < .01). Among 12, 735 network–county combinations, 1.8% failed to meet Medicare's urologist adequacy standards.

**Conclusion:**

MA urology networks vary widely in breadth and composition. Narrow networks are associated with distinct prostate cancer management patterns that may have implications for access and quality, underscoring the need to assess specialty network adequacy standards.

## Introduction

Over the last 2 decades enrollment in Medicare Advantage (MA) has grown steadily, with more than half of all Medicare beneficiaries currently enrolled in MA plans.^1^ The structure of MA—where health plans are reimbursed a capitated rate to cover all services and retain unspent funds as profit—imposes strong incentives for plans to constrain utilization among enrollees.^2^ One mechanism by which MA plans may control costs is through establishment of selective provider networks.^3^ Plans may strategically curate networks by contracting with providers who demonstrate higher quality and/or lower cost performance. As a result, MA networks can vary in breadth and geographic coverage by excluding some portion of providers in a given market.^4^

Selective contracting has been studied in the context of hospitals and nursing homes, as well as for primary care physicians, who serve as the gatekeepers by directing referrals and coordinating care.^5,6^ However, less is known about how selective contracting within MA affects access to specialists—particularly in high-cost, high-volume fields, such as urology. Urologists manage a broad range of costly and prevalent conditions, including benign prostatic enlargement and prostate cancer—the most common cancer among men.^7^ Importantly, treatment of prostate cancer is highly nuanced and costly treatment in the form of surgery or radiation is not always indicated. Similarly, prostate enlargement can often be treated conservatively without requiring a costly surgical procedure. Consequently, MA plans may seek to contract with urologists who are more likely to constrain utilization—for instance, by limiting overtreatment of low-risk prostate cancer in older men. Conversely, narrow networks may unintentionally restrict patient access to high-volume surgeons for complex procedures, such as robotic prostatectomy, with potential implications for quality and outcomes. To our knowledge, the characteristics of urologists participating in MA networks, or how these networks vary in breadth and quality, has not been studied.

For these reasons, we performed a national observational study to characterize the urologists included in MA networks. Specifically, we sought to characterize urologists participating in MA networks by measures of prostate cancer volume and quality. As an additional measure of volume, we assessed the use of benign prostatic hyperplasia (BPH) procedures. We also assessed how these measures varied by network inclusion (ie, in- vs out-of-network) and by breadth of networks (ie, narrow vs broad).

## Data and methods

### Urologist characteristics

Using a 20% sample of traditional Medicare (TM) beneficiaries, we identified 9137 urologists defined as physicians who billed at least 99% of their claims under a urology specialty code. To further ensure accuracy, identified urologists were cross-referenced with the Centers for Medicare and Medicaid Services' (CMS’) Doctors and Clinicians National Downloadable File, yielding 9128 physicians whose primary specialty was listed as urology. Of these urologists, complete demographic data was available for 9124. Urologist patient panel characteristics, including patient volume, percentage of dual-eligible beneficiaries in a practice panel, and the percentage of ethnic minority patients each urologist sees, were identified using claims from all of the patients a urologist treated and the Medicare Beneficiary Summary File. Urologist characteristics including gender and graduation year were obtained from the National Downloadable File. We obtained the average CMS Hierarchical Condition Category (HCC) risk score of each urologist's patient panel from the Medicare Physician and Other Practitioners File.

We defined urology practices and characterized them by practice context (ie, single specialty vs multispecialty); size (eg, number of urologists); TM market concentration, measured by the Herfindahl Hirshman Index (HHI), defined as the sum of the square of the share of each practice's unique patients within the market; and market supply of urologists per 1000 male Medicare beneficiaries. Market concentration for urology practices and market supply were measured at the level of the hospital referral region. Each practice was further characterized as single specialty if all the claims under the tax identification were from urologists or multispecialty if there were claims billed by at least 2 different specialties. Each urologist was assigned to a practice based on the tax identification number under which they billed the plurality of their claims.

### Urologist attributes and measures of volume and quality

We selected several measures of urologist volume that are relevant to prostate cancer. We chose prostate cancer as an exemplary condition given that it is among the most common and expensive conditions urologists manage, particularly among elderly Medicare beneficiaries. Using a 20% sample of TM claims, we pooled data from 2018 and 2021 to obtain sufficient data to define prostate cancer–specific attributes for each urologist, including annual number of prostate biopsies performed and number of prostatectomies (both minimally invasive and open surgical procedures) performed. We used a 2-year look-back period to 2016 to ensure we captured incident cases. To provide an additional reference point, we also quantified annual volume of procedures performed to treat BPH. Given their high prevalence among Medicare beneficiaries, BPH procedures were included as an additional measure of overall urologic procedural volume. Importantly, BPH procedures are similarly costly and discretionary to some degree, which may make them relevant to MA plans' selective contracting decisions. Therefore, this measurement allowed us to assess whether observed patterns of network selection reflected prostate cancer–specific factors or broader differences in urologist procedural activity.

To provide some insight into quality, we assessed 2 quality measures relevant to prostate cancer care that could be reliably calculated using claims, including percentage of initial prostate biopsies preceded by magnetic resonance imaging (MRI) and percentage of beneficiaries with a new diagnosis of prostate cancer who also saw a radiation oncologist.^8,9^ Both of these measures are endorsed by clinical guidelines and expert consensus panels.^10-12^ To calculate the percentage of biopsies that were preceded by an MRI, we identified patients who received biopsies between 2016 and 2018. Next, we determined whether each patient had received a prostate MRI within the 6-month period prior to the biopsy. For all beneficiaries, we maintained a clean 1-year look-back period in order to ensure that cases identified were incident (ie, newly diagnosed) rather than existing cases, which might require different treatment pathways. For the second quality measure, we measured the percentage of Medicare beneficiaries who had a consultation with a radiation oncologist after being diagnosed with cancer. Both the American Urological Association/American Society for Radiation Oncology/American Society of Clinical Oncology and the National Comprehensive Cancer Network guidelines recommend the use of shared decision-making to choose among appropriate treatment options.^8-10,13^ We identified beneficiaries whose first diagnosis of prostate cancer occurred between 2016 and 2018. To ensure these were incident cases, we included a clean 1-year look-back period and required beneficiaries to receive a prostate biopsy within the 6-month period prior to their first (of at least two) E&M (evaluation and management) codes associated with a prostate cancer diagnosis. Finally, we measured the percentage of beneficiaries with an evaluation and management visit with a radiation oncologist within the 6-month period from their initial diagnosis. For both quality measures, we chose to generate our sample with incident measures between 2016 and 2018 so that we could avoid any effects of COVID-19.

### Identifying MA urologist networks

Using 2021 data from Ideon—a firm that compiles MA network data using insurer and plan directories—we identified urologists participating in MA networks.^14^ Networks were defined at the HRR level. To ensure reliability, HRRs with no MA networks and those in US territories were excluded. Furthermore, network HRRs with fewer than 50 beneficiaries or with only 1 urologist were also excluded. We further characterized networks by breadth, based on the percentage of urologists included. Network HRRs were defined as “narrow” if they included fewer than 30% of urologists in a given HRR and “broad” otherwise, based on prior work.^5^

### Statistical analysis

We constructed comparative observations for each MA network–HRR combination by categorizing urologists as either in-network or out-of-network based on the Ideon data. For each HRR, we identified all urologists practicing in that region and, for each MA network operating in the HRR, distinguished the urologists who were included in the plan's network and those who were not. We then calculated average urologist characteristics, prostate cancer–specific practice patterns, and quality measures separately for those in and out of each plan's network within the HRR. This yielded 2 observations for each network HRR: one reflecting network-included urologists and the other representing excluded urologists. Next, we generated HRR- and national-level averages, by calculating enrollee-weighted means across network HRRs, weighting each observation by the number of enrollees in that network HRR. As such, this methodology generates averages that give higher weight to urologists who participate in or are excluded from network HRRs with larger enrollment. Moreover, urologists could contribute to means of both the in-network and out-of-network groups since network inclusion status may differ across carriers.

Using similar methodology as described previously, we constructed comparative observations for narrow and broad networks using parent organization HRR as the unit of analysis. Because urologists may participate in multiple networks within a single parent organization—some broad and some narrow—we classified urologists as participating in a broad network within the parent organization if they exclusively participated in a broad network within a given parent organization. Conversely, we counted a urologist as participating in a narrow network if they participated in at least 1 narrow network within a given parent organization (almost always in addition to the broad network). Next, we calculated average urologist characteristics for each broad and narrow parent organization HRR. Finally, we generated nationally representative averages for urologist characteristics by weighting each broad and narrow parent organization–HRR observation by the proportion of beneficiaries enrolled. Additionally, we conducted 2 stratified analyses: (1) by market concentration (HHI) to distinguish if patterns differed in HRRs with highly concentrated urology practices vs those in less concentrated markets and (2) by the market supply of urologists per 1000 Medicare beneficiaries.

Finally, for each county, we approximated MA network adequacy criteria for the minimum number of urologists required as well as the supply of urologists among MA beneficiaries relative to TM beneficiaries. While the above analysis used HRR as the geographic level of analysis, this assessment focused on counties in order to align with Medicare's guidelines, which define network adequacy standards at the county level. We attributed urologists to a county if they had at least 5 claims in a given county. We then compared the number of urologists in each network–county combination with the minimum number required by CMS for each county.^15^ Next, we quantified relative urologist availability between MA and TM by calculating a county-level ratio of beneficiaries per urologist in a network county to beneficiaries per urologist in TM, where the TM denominator included all urologists attributed to the county. Values greater than 1 indicated lower urologist availability in an MA network relative to TM within the same county. We used Medicare enrollment data to obtain the total number of beneficiaries for both TM and MA in each county.

### Limitations

Findings from this study must be considered in the context of several limitations. First, the assignment of urologists to MA networks relied on Ideon data, which leverages provider directories that could contain inaccuracies, although these data have been verified in other contexts.^5^ Second, measures of provider volume and quality were drawn from a 20% sample of TM patients and may not be fully representative of providers serving predominantly MA enrollees or if urologists tailor treatment by Medicare insurance type. However, because the measurement period (2016–2018) preceded the period of rapid MA expansion, misclassification is likely minimal. Third, volume and quality were assessed for 2 clinical conditions, which may not capture the full scope of urology practice. Nonetheless, we selected these measures because they represent common and costly conditions relevant to most elderly urologic patients covered by Medicare. Fourth, the measures of quality are process rather than outcome measures and cannot account for clinical disease-level characteristics that are not available in claims such as the aggressiveness of the prostate cancer (eg, Gleason score) that would be necessary to assess appropriateness of care at the patient level. Similarly, MRI utilization also may be influenced by geographic availability of MRI in addition to urologist-specific practice patterns.

## Results

**Figure 1.**
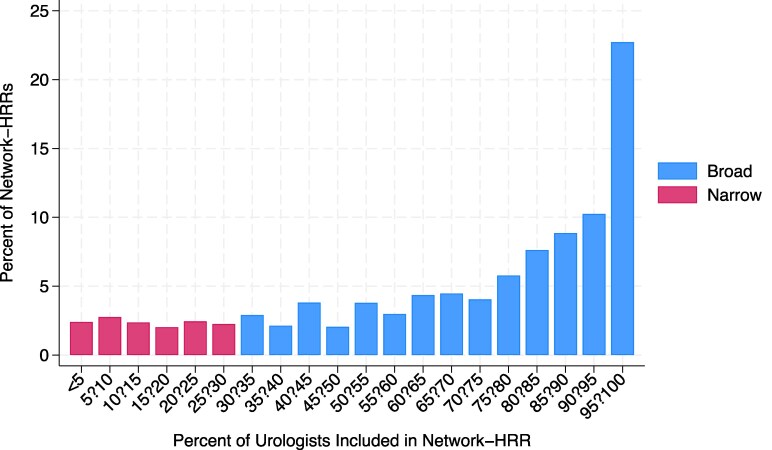
Distribution of the percentage of urologists included within a network HRR, stratified by broad and narrow networks. Source: Authors’ analysis of Ideon Medicare Advantage Network data 2021, 20% sample of traditional Medicare claims 2021, CMS Doctors and Clinicians File 2021, National Downloadable File 2021, and Medicare Physician and Other Practitioners File 2021. Abbreviations: CMS, Centers for Medicare and Medicaid Services; HRR, hospital referral region.

We identified 9124 urologists in 2021 who submitted claims to Medicare. Most urologists (98%) were included in at least 1 MA network. Using data from Ideon, we identified 7516 unique network–HRR combinations. Network HRRs included a mean (SD) of 42 (47) urologists. The proportion of urologists in a given HRR who were included within a network HRR ranged from 9% at the fifth percentile to 100% at the 95th percentile (Figure 1).

### All networks

Compared with the average out-of-network urologist, the average in-network urologist was more likely to be male (90% vs 88%; *P*  < .01), practice in multispecialty groups or larger single-specialty group practices (88% vs 83%; *P*  < .01), have a higher annual patient volume (107 vs 95; *P*  < .01), and have a patient panel with lower HCC scores (1.42 vs 1.44; *P*  < .01) (Table 1).

**Table 1.** Characteristics of urologists and their patients, stratified by in- and out-of-network and by network breadth, weighted by enrollment.

	In-network^a^	Out-of-network^a^		*P* value	Narrow network^b^	Broad network^b^	*P* value
**Urologist characteristics**							
**Female, %**	10	12		<.01	9	10	<.01
**No. of years since graduation**	28	28		.76	28	28	.70
**Practice context, %**							
Single specialty	12	17		<.01	14	12	.03
Multispecialty	88	83		<.01	86	88	.03
**Annual patient volume**, ***n***	107	95		<.01	68	110	<.01
**Patient panel characteristics**							
**Female**, **%**	27	27		<.01	27	26	.06
**Mean age**, **y**	74	73		.18	72	74	<.01
**Race, %**							
White	83	84		<.01	70	84	<.01
Black	8	8		.16	11	8	<.01
Other	9	8		<.01	18	8	<.01
**Dual enrollment**, **%**	16	16		.07	25	15	<.01
Mean HCC* score	1.42	1.44		<.01	1.48	1.41	<.01

Abbreviations: CMS, Centers for Medicare and Medicaid Services; HCC, Hierarchical Condition Category; HRR, hospital referral region; MA, Medicare Advantage.

Source: Authors' analysis of a 20% sample of traditional Medicare claims 2021, CMS Doctors and Clinicians File 2021, National Downloadable File 2021, Medicare Physician and Other Practitioners File 2021, and CMS Medicare Beneficiary Summary File 2021. All patient characteristics were collected first at the physician level before being averaged nationally.

^a^Means were calculated for urologists who participated in MA networks at the network-HRR level, and then averaged nationally, weighted by the number of enrollees using each network HRR.

^b^Means were calculated separately for urologists who participated in any narrow networks (defined as network HRRs including fewer than 30% of urologists in the HRR) and those who participated in exclusively broad networks (defined as network HRRs including 30% or more of urologists in the HRR).

Compared with the average out-of-network urologist, the average in-network urologist performed more prostate biopsies per year (3.18 vs 2.89; *P*  < .01) and performed more radical prostatectomies annually (0.46 vs 0.35; *P*  < .01), including a higher percentage using a minimally invasive approach (89% vs 86%; *P*  < .01) (Table 2). However, there were no differences in the measures of quality between the groups. These results did not vary by market concentration (Table S1) nor urologist supply (Table S2). Similarly, compared with the average out-of-network urologist, the average in-network urologist performed more total BPH procedures (Table S3).

**Table 2.** Prostate cancer measures of volume and quality, stratified by in- and out-of-network and by network breadth, weighted by enrollment.

	In-network^a^	Out-of-network^a^	*P* value	Narrow network^b^	Broad network^b^	*P* value
Annual No. of prostate biopsies	3.18	2.89	<.01	2.15	3.29	<.01
Annual No. of radical prostatectomies	0.46	0.35	<.01	0.24	0.49	<.01
Prostatectomies performed minimally invasively, %	89	86	<.01	90	89	.37
Newly diagnosed patients with prostate cancer who underwent prostatectomy, %	14	13	<.01	21	14	<.01
Prostate biopsies preceded by pelvic MRI, %	12	11	.03	15	12	<.01
Patients with new diagnosis of prostate cancer who saw a radiation oncologist, %	54	54	.63	50	54	<.01

Abbreviations: HRR, hospital referral region; MA, Medicare Advantage; MRI, magnetic resonance imaging.

Source: Authors' analysis of a 20% sample of traditional Medicare Claims 2016 to 2019. All patient-level measures were collected first at the physician level before being averaged nationally.

^a^Means were calculated for urologists who participated in MA networks at the network-HRR level, and then averaged nationally, weighted by the number of enrollees using each network HRR.

^b^Means were calculated separately for urologists who participated only in narrow networks (defined as network HRRs including fewer than 30% of urologists in the HRR) and broad (defined as network HRRs including 30% or more of urologists in the HRR).

### Narrow vs broad networks

When stratified by network breadth, urologists included in narrow networks were more likely to practice in single-specialty groups (14% vs 12%; *P*  < .01), have a lower annual patient volume (68 vs 110; *P*  < .01), serve more dual-eligible beneficiaries (25% vs 15%; *P*  < .01), and care for patient panels with higher HCC scores (1.48 vs 1.41; *P*  < .01). Narrow-network urologists also performed fewer prostate biopsies (2.15 vs 3.29; *P*  < .01) and fewer radical prostatectomies (0.24 vs 0.49; *P*  < .01), although the percentage performed using a minimally invasive approach was similar (90% vs 89%; *P* = .37). In contrast, they were more likely to perform prostatectomy for beneficiaries with a new prostate cancer diagnosis (21% vs 14%; *P*  < .01).

When comparing measures of quality, beneficiaries managed by narrow-network HRR urologists were more likely to obtain an MRI prior to prostate biopsy (15% vs 12%; *P*  < .01), but less likely to see a radiation oncologist following a new diagnosis of prostate cancer (50% vs 54%; *P*  < .01).

These results varied by market concentration (Table S1) and urologist supply (Table S2). Specifically, in areas of lower market concentration, urologists in narrow networks were more likely to perform prostatectomy for beneficiaries with a new prostate cancer diagnosis (22% vs 14%; *P*  < .01), but this effect was not observed in areas of high market concentration (13% vs 12%; *P* = .66). We observed a similar finding comparing urologists in narrow vs broad networks in areas of high urologist supply (26% vs 14%; *P*  < .01). This effect was not observed in areas of low urologist supply (10% vs 13%; *P*  < .01). These findings were similar for BPH procedures (Table S3).

### Network adequacy

**Figure 2.**
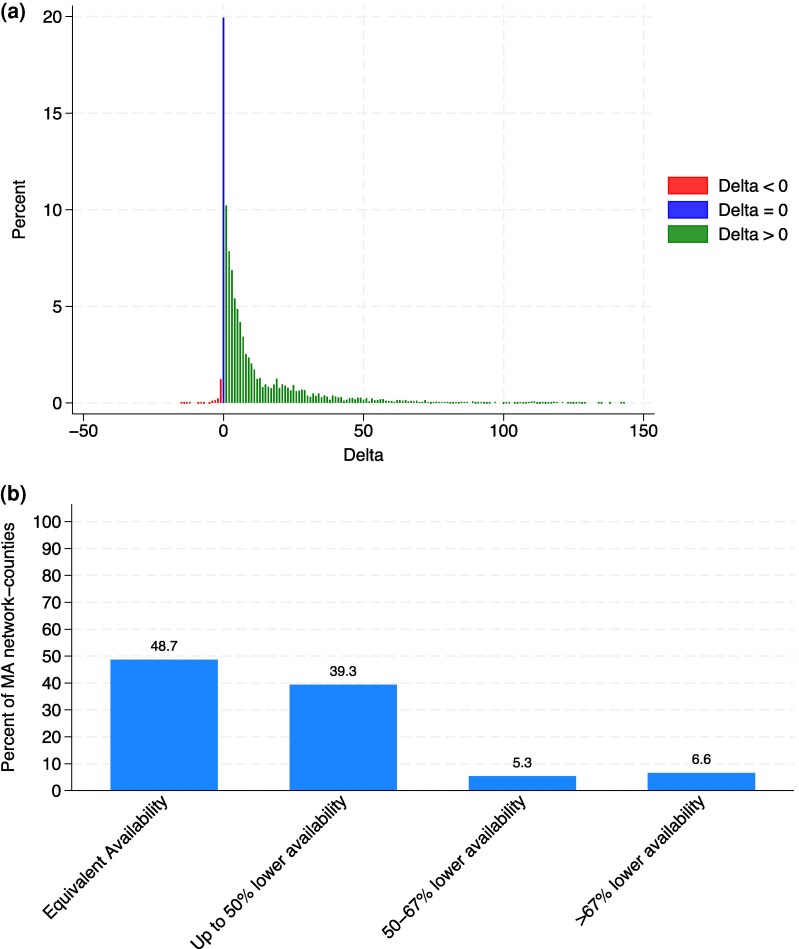
(a) Distribution of the difference between network–county urologist count and Medicare's adequacy requirement (Delta). (b) Comparison of urologist availability between TM and MA networks at the county level. Source: Authors’ analysis of CMS adequacy criteria, Ideon Medicare Advantage Network data 2021, CMS Doctors and Clinicians File 2021, National Downloadable File 2021, and Medicare Physician and Other Practitioners File 2021. Bars in panel B represent the percentage of networks in each availability category (ie, 39.3% of network counties have between 1 and 2 times more beneficiaries per urologist). Abbreviations: CMS, Centers for Medicare and Medicaid Services; MA, Medicare Advantage; TM, traditional Medicare.

Finally, we measured how many network–county combinations met Medicare's requirements, which stipulate the minimum number of providers, by specialty, that must be in-network in each county. Among the 12 735 network–county combinations, 1.8% did not meet the adequacy criteria for the minimum number of urologists, 20% met the criteria precisely, while 78% exceeded the criteria (Figure 2a). Compared with TM, 49% of MA network counties demonstrated equivalent urologist availability. Among the remainder, 39.3% had up to 50% lower availability and 11.9% had more than 50% lower availability (Figure 2b).

## Discussion

In this national study, we found substantial variation in the inclusion of urologists across MA networks by region and network breadth. Although most urologists participated in at least 1 MA network, both the share and characteristics of included providers varied widely. In particular, urologists included in more and larger MA networks had higher procedural volume compared with those often excluded from networks, without corresponding differences in measured prostate cancer care quality. The average urologist included in narrow networks had overall less volume but was more likely to perform prostatectomy among men diagnosed with prostate cancer, compared with the average urologist included broad networks. Prior work has demonstrated selective contracting among primary care physician^5^; these findings suggest that MA plans may also selectively contract with specialty providers such as urologists. While volume may represent 1 dimension along which selection occurs, selective contracting may also reflect other factors, including costs, practice infrastructure, or market characteristics, that are not measured in this study.

We observed the most significant differences when examining urologists participating in narrow vs broad networks. Those included in narrow networks were more often affiliated with smaller group practices, treated medically complex patients, and had overall lower procedure volumes. Despite lower volume, they were more likely to perform surgery for newly diagnosed prostate cancer, particularly in less-concentrated markets or those with high urologist supply. These findings align with prior work demonstrating higher treatment rates in less-concentrated markets, even among men who may not benefit due to competing mortality risks.^16^ Quality was mixed between narrow- and broad-network urologists. Patients managed by narrow-network urologists were less likely to receive a consultation with radiation oncology, potentially suggesting limited access to multidisciplinary care. However, they were more likely to obtain an MRI prior to prostate biopsy, a practice associated with reduced detection of low-risk disease (ie, the form of prostate cancer that most often does not require treatment).^17^ These findings suggest that narrow-network urologists may exhibit more selective diagnostic practices but greater reliance on surgical treatment once prostate cancer is diagnosed. Surgery is generally less expensive than radiation, which may partly explain this pattern in the context of MA plans' cost-containment incentives.^18^

Collectively, these differences between narrow- and broad-network urologists may reflect intersecting influences of market pressures, referral pathways within networks, and financial incentives on clinical decision-making.^16^ Such variation is especially consequential for prostate cancer, where the decision to treat can be discretionary and sensitive to nonclinical factors.^19,20^ Prostate cancer management spans a spectrum of options, including active surveillance, surgery, and radiation. Among these, radiation is the most costly, followed by surgery, with active surveillance being the least-expensive option, particularly in the first year after diagnosis.^18^ In this setting, higher rates of surgical treatment among narrow-network urologists may be partially consistent with MA plans' objective of constraining costs. Active surveillance, although the least-costly strategy upfront, requires longitudinal follow-up, ongoing diagnostic testing, and access to multidisciplinary care. These requirements may be less readily supported within narrow networks characterized by smaller practices. Consequently, MA plans' reliance on restrictive specialty networks and directed referrals may shape treatment decisions in ways that reflect network design more than clinical need, potentially contributing to unnecessary treatment of a common and costly disease. Taken together, these findings raise concern that current approaches to specialty network construction may not adequately account for guideline-concordant management strategies for conditions such as prostate cancer.

Although nearly all MA networks met Medicare's adequacy criteria for urologist inclusion, the proportion of urologists participating within counties varied widely, with some plans including as few as 9% of urologists within a given county. This aligns with prior evidence demonstrating variation in network breadth across specialities.^4^ Beyond individual provider characteristics, these findings raise broader concerns about how network adequacy is conceptualized and regulated. Current adequacy standards focus on provider counts and time-distance thresholds, yet our results show that networks can meet these benchmarks while differing markedly in composition and measures of quality. Moreover, adequacy thresholds that treat all providers as interchangeable may obscure important differences in surgical expertise.^21,22^ Consistent with this concern, men managed by urologists in narrow networks are more likely to undergo surgery with lower-volume providers—a characteristic that prior work has linked to worse patient-reported and oncologic outcomes.^21^ Therefore, in the context of surgical specialty care, these findings raise concerns that existing network adequacy requirements may overlook clinically important variation in access to high-quality surgeons.

Our findings have policy-relevant implications that extend beyond urology to other specialty care. Selective contracting in MA may not be confined to primary care and may substantially influence the configuration of networks for other specialties, particularly where high procedural costs and discretionary treatment decisions may make selective contracting most consequential. Medicare Advantage plans have financial incentives to partner with providers and hospitals that deliver margin-preserving efficiencies. However, these contracting patterns could constrain access to surgical expertise and multidisciplinary services for complex conditions. Accordingly, current standards of network adequacy may warrant reconsideration in the context of specialty care.

In summary, MA urology networks vary not only in breadth but also in the characteristics of included urologists. As MA enrollment continues to grow, greater scrutiny of specialty network design and its implications for access and quality is warranted.

## Supplementary Material

qxag172_Supplementary_Data

## Data Availability

The data used in this study were obtained under a data use agreement with the Centers for Medicare & Medicaid Services (CMS) and cannot be publicly shared or made available due to the terms of that agreement. Researchers seeking access to comparable Medicare data may apply directly to CMS through the Research Data Assistance Center (ResDAC, https://resdac.org). Code used for data management and analysis is available from the corresponding author upon reasonable request.
