# Supplementary material for: Characteristics of urologists participating in Medicare Advantage networks and implications for prostate cancer care
Source: Health Aff Sch. 2026 Jul 4;4(7):qxag172. doi: 10.1093/haschl/qxag172 (PMC13404993; doi:10.1093/haschl/qxag172)
Supplement: qxag172_Supplementary_Data [file qxag172_supplementary_data.zip › Appendix (4).docx]

**Appendix**

**Supplemental Table 1:** Prostate cancer measures of volume and quality, stratified high vs. low HHI, by in- and out-of-network and by network breadth, weighted by enrollment.

**Supplemental Table 2:** Prostate cancer measures of volume and quality, stratified high vs. low urologist supply, by in- and out-of-network and by network breadth, weighted by enrollment.

**Supplemental Table 3:** BPH procedure stratified by in- and out-of-network and by network breadth, weighted by enrollment.

**Supplemental Table 1**. Prostate cancer measures of volume and quality, stratified high vs. low HHI, by in- and out-of-network and by network breadth, weighted by enrollment.

|  | High HHI | |  | Low HHI | |  |
| --- | --- | --- | --- | --- | --- | --- |
|  | ***In-Network^a^*** | ***Out-of-Network^a^*** | ***p-value*** | ***In-Network^a^*** | ***Out-of-Network^a^*** | ***p-value*** |
| Annual number of prostate biopsies | 3.74 | 2.90 | <0.01 | 2.99 | 2.88 | <0.01 |
| Annual number of radical prostatectomies | 0.45 | 0.33 | <0.01 | 0.46 | 0.36 | <0.01 |
| Percentage of prostatectomies performed minimally invasively (%) | 92 | 89 | <0.01 | 89 | 85 | <0.01 |
| Percent of newly diagnosed prostate cancer patients that underwent prostatectomy (%) | 13 | 12 | 0.13 | 15 | 14 | <0.01 |
|  |  |  |  |  |  |  |
| Percent of prostate biopsies preceded by pelvic MRI | 11 | 11 | 0.39 | 12 | 12 | 0.08 |
| Percent of patients with new diagnosis of prostate cancer who saw a radiation oncologist | 55 | 55 | 0.57 | 53 | 53 | 0.98 |
|  | ***Narrow Network^b^*** | ***Broad Network^b^*** | ***p-value*** | ***Narrow Network^b^*** | ***Broad Network^b^*** | ***p-value*** |
| Annual number of prostate biopsies | 3.02 | 3.75 | <0.01 | 2.03 | 3.12 | <0.01 |
| Annual number of radical prostatectomies | 0.17 | 0.46 | <0.01 | 0.25 | 0.49 | <0.01 |
| Percentage of prostatectomies performed minimally invasively (%) | 52 | 93 | <0.01 | 94 | 88 | <0.01 |
| Percent of newly diagnosed prostate cancer patients that underwent prostatectomy (%) | 12 | 13 | 0.66 | 22 | 14 | <0.01 |
|  |  |  |  |  |  |  |
| Percent of prostate biopsies preceded by pelvic MRI | 8 | 11 | <0.01 | 16 | 12 | <0.01 |
| Percent of patients with new diagnosis of prostate cancer who saw a radiation oncologist | 47 | 55 | <0.01 | 51 | 54 | 0.02 |

^a^ Means were calculated for urologists who participated in MA networks at the network-HRR level, and then averaged nationally, weighted by the number of enrollees using each network-HRR.

^b^ Means were calculated separately for urologists who participated only in narrow networks (defined as network-HRRs including fewer than 30% of urologists in the HRR) and broad (defined as network-HRRs including 30% or more of urologists in the HRR).

**Supplemental Table 2**. Prostate cancer measures of volume and quality, stratified high (above overall median) vs. low (below overall median) urologist supply, by in- and out-of-network and by network breadth, weighted by enrollment.

|  | High Supply | |  | Low Supply | |  |
| --- | --- | --- | --- | --- | --- | --- |
|  | ***In-Network^a^*** | ***Out-of-Network^a^*** | ***p-value*** | ***In-Network^a^*** | ***Out-of-Network^a^*** | ***p-value*** |
| Annual number of prostate biopsies | 2.95 | 2.64 | <0.01 | 3.56 | 3.27 | <0.01 |
| Annual number of radical prostatectomies | 0.46 | 0.37 | <0.01 | 0.44 | 0.32 | <0.01 |
| Percentage of prostatectomies performed minimally invasively (%) | 88 | 86 | <0.01 | 92 | 85 | <0.01 |
| Percent of newly diagnosed prostate cancer patients that underwent prostatectomy (%) | 15 | 14 | <0.01 | 13 | 12 | <0.01 |
|  |  |  |  |  |  |  |
| Percent of prostate biopsies preceded by pelvic MRI | 13 | 13 | 0.24 | 10 | 9 | 0.07 |
| Percent of patients with new diagnosis of prostate cancer who saw a radiation oncologist | 55 | 55 | 0.28 | 52 | 52 | 0.73 |
|  | ***Narrow Network^b^*** | ***Broad Network^b^*** | ***p-value*** | ***Narrow Network^b^*** | ***Broad Network^b^*** | ***p-value*** |
| Annual number of prostate biopsies | 1.88 | 3.05 | <0.01 | 2.61 | 3.67 | <0.01 |
| Annual number of radical prostatectomies | 0.30 | 0.48 | <0.01 | 0.15 | 0.49 | <0.01 |
| Percentage of prostatectomies performed minimally invasively (%) | 90 | 88 | 0.19 | 94 | 92 | 0.37 |
| Percent of newly diagnosed prostate cancer patients that underwent prostatectomy (%) | 26 | 14 | <0.01 | 10 | 13 | <0.01 |
|  |  |  |  |  |  |  |
| Percent of prostate biopsies preceded by pelvic MRI | 14 | 13 | 0.12 | 16 | 9 | <0.01 |
| Percent of patients with new diagnosis of prostate cancer who saw a radiation oncologist | 49 | 55 | <0.01 | 53 | 52 | 0.64 |

^a^ Means were calculated for urologists who participated in MA networks at the network-HRR level, and then averaged nationally, weighted by the number of enrollees using each network-HRR.

^b^ Means were calculated separately for urologists who participated only in narrow networks (defined as network-HRRs including fewer than 30% of urologists in the HRR) and broad (defined as network-HRRs including 30% or more of urologists in the HRR).

**Supplemental Table 3.** BPH procedure stratified by in- and out-of-network and by network breadth, weighted by enrollment.

|  | In-Network^a^ | Out-of-Network^a^ | p-value | Narrow Network^b^ | Broad Network^b^ | p-vaule |
| --- | --- | --- | --- | --- | --- | --- |
| TURP | 1.11 | 0.99 | <0.01 | 0.78 | 1.13 | <0.01 |
| Laser vaporization | 0.35 | 0.29 | <0.01 | 0.20 | 0.37 | <0.01 |
| HOLEP | 0.12 | 0.11 | 0.08 | 0.05 | 0.13 | <0.01 |
| TUMT/TUNA | 0.04 | 0.03 | 0.11 | 0.02 | 0.04 | <0.01 |
| Urolift | 0.62 | 0.52 | <.01 | 0.38 | 0.63 | <0.01 |
| Simple prostatectomy | 0.03 | 0.03 | 0.69 | 0.01 | 0.03 | <0.01 |
| Water vaporization | 0.19 | 0.16 | <0.01 | 0.11 | 0.20 | <0.01 |
| Total | 2.47 | 2.14 | <0.01 | 1.55 | 2.53 | <0.01 |

^a^ Means were calculated for urologists who participated in MA networks at the network-HRR level, and then averaged nationally, weighted by the number of enrollees using each network-HRR.

^b^ Means were calculated separately for urologists who participated only in narrow networks (defined as network-HRRs including fewer than 30% of urologists in the HRR) and broad (defined as network-HRRs including 30% or more of urologists in the HRR).

Abbreviations: TURP; transurethral resection of prostate, HOLEP; holmium laser enucleation of prostate, TUMT; transurethral microwave ablation therapy, TUNA; transurethral needle ablation
